# Patients with oral lichen planus display lower levels of salivary acidic glycoproteins than individuals without oral mucosal disease

**DOI:** 10.1007/s00784-023-05411-6

**Published:** 2023-12-20

**Authors:** H. Çevik‐Aras, Shehed Musa, Richard Olofsson, Annica Almståhl, Ulrica Almhöjd

**Affiliations:** 1 Department of Oral Medicine and Pathology, Institute of Odontology, University of Gothenburg, Gothenburg, Sweden; 2 Specialist Clinic for Orofacial Medicine, Northern Älvsborg County Hospital, Public Dental Service, Trollhättan, Region Västra Götaland Sweden; 3 Public Dental Service, Gothenburg, Region Västra Götaland Sweden; 4 Specialist Clinic for Orofacial Medicine, Public Dental Service, Uddevalla-Trollhättan, Region Västra Götaland Sweden; 5 Section 4, -Oral Health, Faculty of Odontology, Malmö University, Malmö, Sweden; 6 Department of Oral Microbiology and Immunology, Institute of Odontology, University of Gothenburg, Gothenburg, Sweden; 7 Department of Cariology, Institute of Odontology, University of Gothenburg, Gothenburg, Sweden

**Keywords:** Calcium, Dry mouth, Mucins, Oral premalignant diseases, Total protein

## Abstract

**Objectives:**

Salivary proteins, acidic glycoproteins, and free calcium might take part in oral mucosal defence against inflammation in oral lichen planus (OLP). The study aimed to investigate whether the levels of sulfated and sialylated glycoproteins, total protein, and free calcium in saliva from patients with OLP differ from those of individuals without oral mucosal diseases.

**Material and Methods:**

Patients diagnosed with OLP (*n* = 25) and two control groups without any oral mucosal disease; age- and gender-matched controls (*n* = 25, 65.6 ± 2.9 years), and younger controls (*n* = 25, 41.8 ± 2.5 years) were included. Subjective dry mouth (xerostomia) was assessed by asking a single-item question. Chew-stimulated whole saliva was collected to measure sulfated and sialylated glycoproteins by the Alcian Blue method. The total protein was determined spectrophotometrically, and the free calcium measured using an electrode.

**Results:**

The output of salivary sulfated and sialylated glycoproteins in the OLP group (21.8 ± 2.4 µg/min) was lower than in the age- and gender-matched controls (43.0 ± 2.9 µg/min, *p* = 0.0002), whereas the total protein and calcium output did not differ between the three groups (p > 0.05). The prevalence of xerostomia was significantly higher in the OLP group compared to both control groups (*p* = 0.038).

**Conclusions:**

Patients with OLP showed a high prevalence of xerostomia and lower levels of salivary acidic type glycoproteins compared to the individuals without oral mucosa disease.

**Clinical relevance:**

It is relevant to investigate the role of acidic glycoproteins in the pathogenesis of OLP.

## Introduction

Glycoproteins are rich in negatively charged groups of sialic acids and sulfate residues that contribute to the rheological properties of the saliva [1, 2]. Mucins are large glycoproteins and may contain one or more negatively charged sialic acid (N-acetylneuraminic acid, sialomucins) and/or sulfated sugars (SO_3_GlcNAc, sulfomucin). Sulfated and sialylated units with strong anionic character strengthen the water-holding capacity of the mucin molecule and its interaction with hard and soft tissues [3]. Thus, the structural properties of glycoproteins have roles in the immune defence, water-holding ability and resistance against proteases [2]. As a result, they affect the physicochemical and functional qualities of the saliva, including viscosity, lubrication, and bacterial clearance [2–4].

Acidic types of glycoproteins have been identified in the salivary glands and in the secretory glands of the respiratory and gastrointestinal systems [5]. However, the sialic acid and sulfate content varies largely depending on the mucosal tissue where they are expressed. In saliva and colon, highly sulfated mucins predominate, whereas in the stomach and small intestine, low sulfated mucins are predominant [6]. Salivary mucins, such as MUC5B, MUC7, and MUC1, are abundant in sulfate and sialic acid residues and are primarily found in the mucous secretions of the submandibular, sublingual, and minor salivary glands [6, 7]. However, in addition to mucins, saliva also contains other less complex glycoproteins that also contain sulfate and sialic acid residues, such as agglutinin, proline-rich proteins, statherins, and histatins, which are present in serous secretions from the parotid gland [6]. All these proteins interact with oral epithelial cells, creating a protective coating [8]. The acidic glycoproteins present in this coating can modulate the calcium channel activity as shown by Slomiany et al*.* in rat buccal mucosal cells [9]. In the cell, in response to a chemical, electrical, or physical stimulus, intracellular calcium concentrations rise from an influx of extracellular or intracellular calcium stores [10]. The expression of human beta-defensin 2 in gingival epithelial cells has been shown to increase by elevated calcium levels from intracellular and/or extracellular stores [11]. Previous studies have also shown that calcium participates in diverse functions in the oral cavity, including cell growth, proliferation, cell death, and as well as immune response [9, 11–15]. Moreover, calcium is a key mediator of mucin network organization as shown for MUC5B in human whole saliva [16].

Oral lichen planus (OLP) is a chronic inflammatory disorder of the oral mucosa, which affects 1–2% of the global population [17]. The pathogenesis of OLP is still not fully known; but both specific and non-specific immune systems are suggested to be involved [18]. Unknown antigens on the cell surface of oral keratinocytes have been suggested to initiate T cell activation, followed by apoptosis of the keratinocytes [18]. In a previous cross-sectional study, lower flow rates for unstimulated and stimulated saliva have been found in patients with OLP compared to the control group [19]. In the same study, lower levels of M3 muscarinic receptors have been shown [19]. Moreover, increased levels of pro-inflammatory cytokines were reported in the saliva of patients with OLP [20]. There are several studies supporting the idea that alterations in the acidic type of glycoproteins are involved in inflammatory conditions as well as in malignancies [21, 22]. The total protein concentration was shown to be lower in the unstimulated saliva, whereas it was higher in the chewing-stimulated saliva of patients with OLP when compared to healthy controls [23]. Few studies in patients with OLP showed confliction results regarding calcium levels [24–26]. However, acidic glycoproteins and their levels in saliva have yet to be studied in patients with OLP.

Microscopically, saliva is composed of clusters of particles called salivary micelles, which are formed by the intermolecular interactions of glycoproteins, other small proteins, and ions [27]. The measurement of salivary glycoproteins is challenging and time-consuming due to their complex structure, which requires the use of various methods including, chromatography and mass spectrometry [28]. Alcian Blue is a cationic dye widely used in light microscopy to stain sulfated and sialic acids containing acidic type glycoproteins [29]. The method has later been optimised for quantitative measurement of intestinal mucous glycoproteins by a simple biochemical method [30]. In the present study, the same method was used to analyse saliva with a few modifications.

The hypothesis of the study was that salivary proteins, acidic glycoproteins, and free calcium could take part in oral mucosal defence, and measurement of their levels can be used to investigate their association with OLP. Accordingly, the aim of the present study was to investigate whether the levels of sulfated and sialylated glycoproteins, total protein, and free calcium in chew-stimulated saliva from patients with OLP differ from those of individuals without oral mucosal disease.

## Materials and methods

### Participants

A detailed medical history of all study participants was recorded, including information on diseases, medications, oral symptoms, allergies, alcohol consumption, use of snuff, and smoking. Additionally, all study participants were asked a single-item question: "Have you experienced dry mouth in the last six months?" Patients who answered "No" to this question were considered to not have subjective dry mouth [31]. The study was performed in accordance with the Declaration of Helsinki. The Central Ethical Review Board in Gothenburg, Sweden, approved the study (permit number T-512/18) and written informed consent was obtained from all individuals in the study.

Twenty-five patients referred to the Specialist Clinic for Orofacial Medicine (Northern Älvsborg County Hospital, Trollhättan, Sweden), and who fulfilled the clinical and histopathological criteria for the diagnosis of OLP (*n* = 25) [32] were included in the study. Punch biopsies were taken by author HCA, as well as oral swab culture testing (E-swab, Nordic Biolabs AB, Sweden) for the diagnosis of fungal infections.

Age- and gender-matched controls (*n* = 25) with no history of oral mucosal lesions and/or any type of cutaneous lichen planus or lichen sclerosis were included. These patients were referred to the same Specialist Clinic for oral examinations for other reasons than oral mucosal lesions and the oral examinations were performed by author HCA. Additionally, a young control group aged 22–50 years (*n* = 25) was recruited from volunteering students and employees at the Institute of Odontology, University of Gothenburg. The recruitment and the oral examination of younger control group were performed by authors HCA and SM. This control group had no oral or cutaneous lesions, diseases, or medication. Furthermore, they had no smoking habits and did not report subjective dry mouth.

### Collection and handling of saliva samples

All individuals in the study were instructed to refrain from eating, drinking, and performing oral hygiene procedures for at least one hour prior to saliva collection, which was performed between 9 am—12 am to minimise diurnal variations. To collect chew-stimulated saliva, the participants were asked to chew on a paraffin piece to gather saliva in the mouth, which was spat out into a 10-ml tube until 3 ml had been collected. The sampling time was approximately three minutes [33]. The saliva volume was measured, and the salivary flow rate (ml/min) calculated. Protease inhibitor cocktail tablets (Sigma-Aldrich, S8830; one tablet diluted in 4 ml distilled water 25 µl/ml were used) and EDTA (Sigma-Aldrich, 2 mM) were immediately added to minimise protein degradation [34]. The samples were stored at − 70 °C until analysis.

### The Alcian Blue method

The Alcian Blue method was used to measure the amount of sulfated and sialylated glycoproteins in stimulated saliva. The method has previously been used to measure intestinal mucous glycoproteins [30] and was applied to the saliva with few modifications, as described below.

**Fig. 1:**
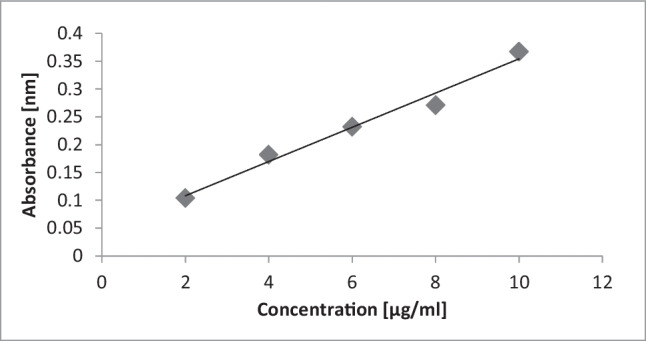
Standard curve obtained by chondroitin sulfate (0.2 mg/ml) for the quantitative measurement of sulfated and sialylated glycoproteins

A solution of 0.1 w/v (weight/volume) Alcian Blue in 0.1 M sodium acetate acid buffer (pH 5.8) containing 25 mM MgCl_2_ was used. Chondroitin sulfate was used to obtain a standard curve for the sulfated and sialylated glycoproteins [30]. A five-point concentration gradient with the highest concentration of 0.2 mg/ml chondroitin sulfate was used to create the absorbance-concentration curve. The absorbance was measured by a spectrophotometer at 620 nm (Fig. 1).

Chondroitin sulfate (0.2 mg/ml, 50 µl) and saliva samples (50 µl) were diluted in 1:3 with Krebs–Henseleit bicarbonate buffer without glucose (100 µl; 118 mM NaCl, 4.7 mM KCl, 1.2 mM MgSO_4_, 1.25 mM CaCl_2_, 1.2 mM KH_2_PO_4_, 25 mM NaHCO_3_) and incubated for at least 30 min. at room temperature to improve dissolution of the glycoproteins. Fifty µL Alcian Blue was then added to each analyte to a final volume of 200 µL, whereafter the analytes were incubated for 30 min at room temperature to allow the sulfated and sialylated glycoproteins to form complexes with the Alcian Blue solution. The samples were then centrifuged at 3000 rpm for 15 min. at 20 ºC. The supernatants were then removed, and each pellet was washed twice in 0.1 M sodium acetate buffer (pH 5.8) containing 25 mM MgCl_2_ and precipitated at 4000 rpm at 20 ºC for 10 min. The supernatants were then discarded, and the pellet was dissolved in 0.9% sodium chloride solution that was sonicated for 10 min. at 50 ºC. Finally, the properly dissolved analytes were measured at 620 nm in the spectrophotometer [30]. The concentration of sulfated and sialylated glycoproteins (µg/ml) was determined according to the standard curve obtained with chondroitin sulfate (0.2 mg/ml).

### Standard curve for the Alcian Blue method

A linear correlation between the absorbance and concentration of chondroitin sulfate was achieved at 0.2 mg/ml, which means that the absorbance value is positively correlated with increased concentrations of sulfated and sialylated glycoproteins (Fig. 1).

### Intra-assay variation for Alcia Blue method

Three replicates of twelve saliva samples were analysed in a single assay to determine the intra-assay variation. The intra-assay variation (%) for each sample was calculated using the formula: (standard deviation of concentration) / (mean of concentration) × 100.

### Analysis of the total protein concentration

The total protein concentration was analysed using the BCA Protein Assay Kit (Pierce, Rockford, IL, USA) with bovine serum albumin (BSA) as a standard. The saliva samples were diluted with distilled water to 1:2 and 1:4. All samples were analysed in duplicates. After addition of reagents as described by the manufacturer, the plate was incubated for 30 min at 37 °C. The plate was thereafter read at 562 nm in an ELISA reader (Synergy 2, Biotech, Highland Park, Winooski). Total protein concentration was determined (mg/ml).

### Calcium measurement

Free (dissolved) calcium concentration in chew-stimulated saliva was determined by using a calcium electrode (Thermo Fisher Scientific, MA, USA). The accuracy of the electrode was executed by a standard curve using the water hardness standard (Orion ionplus®, application solution, Orion 923206, ThermoScientific) 100 ppm as CaCO_3_ in serial dilution (5x, 10x, 50 × and 100x) with 2% Ionic Strength Adjuster (ISA, Orion ionplus®, application solution, Orion 9232011, ThermoScientific) in accordance with the kit’s instructions.

A two-point calibration was performed by a tenfold dilution (100 and 10 ppm) reusing the beforementioned water hardness standards prior to the direct measurements of the saliva. Samples must be in the linear range of the electrode giving a slope of (-27mv). The following direct calcium concentration of the saliva (800 µl) mixed with (200 µl) ISA was thereafter recorded in ppm. Tests were run at three different time points and a mean value was calculated.

In addition, examining the revealed ppm measurements from mV values into in mol/L, two internal standards of both CaCl_2_ and CaCO_3_ in ultra-pure water ranging from 10^–1^ mol/L to 10^–5^ mol/L were performed for the conversion of mv to mol/L by two different STD curves. (See supplementary files).

### Saliva secretory output

As the capacity to secrete saliva varies greatly between individuals, the total secretory output of glycoproteins (µg/min), total proteins (mg/min), and calcium were calculated by multiplying the salivary flow rate (ml/min) for the respective subject by the concentration of sulfated and sialylated glycoproteins (µg/ml), total protein (mg/ml) and calcium [34].

### Statistics

Univariate analysis, including an unpaired t-test and ordinary one-way ANOVA, was performed using GraphPad Prism (version 9.0.0, GraphPad Software, La Jolla, CA, USA) to compare the levels of sulfated and sialylated glycoproteins, total protein, calcium and salivary flow rates in patients with those of the controls.

Chi-square and Fisher´s exact (2-sided) tests were used for comparison of subjective dry mouth between the OLP group and the age- and gender-matched controls. A *p* value < 0.05 was regarded as being statistically significant.

## Results

### Participants

In this cross-sectional study, a group of patients with OLP (*n* = 25) was compared with age- and gender-matched controls (mean age 66 years, *n* = 25). Additionally, a control group with younger individuals (mean age 42 years, *n* = 25) was also included to investigate the potential impact of age and medications on the salivary secretion of sulfated and sialylated glycoproteins, total protein, and calcium.

Table 1 shows the demographic data of the study groups and the clinical characteristics of patients with OLP. A high proportion both in the OLP group (84%) and in the gender-matched control group (64%) used medicines, mostly for hypertension or hyperlipidemia (OLP *n* = 11 and age- and gender-matched control group (*n* = 9). In addition, the OLP group had individuals diagnosed with other conditions, such as type 2 diabetes (*n* = 10), genital lichen planus (*n* = 3), fibromyalgia (*n* = 1), psoriasis arthritis (*n* = 1), asthma (*n* = 1), depression (*n* = 1), and hypothyroidism (*n* = 1). Smoking was rare in both the OLP group (*n* = 2) and the age- and gender-matched control group (*n* = 1). The symptoms observed in patients with OLP were pain, burning sensation of the oral mucosa, sensitivity to hot and spicy foods, dry mouth, discomfort when speaking, chewing, and tooth brushing.

**Table 1:** Demographics of the participants and the clinical characteristics of the patients with OLP

	OLP group	Age- and gender matched control group	Younger control group
*Number of participants*	25	25	25
*Gender*			
*Number of females*	14	14	16
*Number of males*	11	11	9
*Age (years, mean* ± *SEM)*	66.5 ± 2.3	65.6 ± 2.9	41.8 ± 2.5
*Number of smokers*	2	1	None
*Medications*	84% (*n* = 21)	64% (*n* = 16)	None
*Clinical characteristics of OLP*			
*Atrophic Erosive*	76% 24%	None	None
*Single lesions* *Multiple lesions*	20% 80%	None	None
*Localization of lesions*			
*Buccal mucosa* *Gingiva* *Tongue* *Labial mucosa*	80% 48% 32% 20%	None	None
*Symptom*			
*Moderate pain* *Severe pain*	64% 36%	None	None
*Fungal infections* *Xerostomia*	88% 80%	None 48%	None None

Patients with OLP presented mostly with the atrophic form (*n* = 19, 76%). Reticular lesions of OLP were also observed in patients representing atrophic (*n* = 7) and erosive (*n* = 3) forms. Twenty patients (80%) had oral lesions at multiple sites. The most common site was the buccal mucosa (*n* = 20) (Table 1). All the patients with OLP moderate (*n* = 16) or severe (*n* = 9) pain. Fungal infections were present in most of the patients with OLP. *Candida albicans* was found in 22 of the patients. One patient with atrophic OLP had growth of both *Candida albicans* and *Candida parapsilosis*.

Subjective dry mouth was prevalent among the patients in the OLP group (*n* = 20, 80%), with a very high intake of medications (*n* = 21, 84%). Similarly, the age- and gender-matched control group also reported complaints of subjective dry mouth (*n* = 12, 48%) with a relatively high medication intake (*n* = 16, 64%). Xerostomia was significantly more prevalent in the OLP group than in the age- and gender-matched controls (*p* = 0.038).

Stimulated saliva flow rates varied greatly. In the control groups (*n* = 50), the highest salivary flow rate was 4.8 ml/min, while the lowest was 0.5 ml/min. In the OLP group, the highest flow rate observed was 3.3 ml/min, and the lowest was 0.3 ml/min. The OLP group demonstrated a significantly lower salivary flow rate, with a mean of 1.5 ml/min, compared to both the age- and gender-matched controls and the young control group (with means of 2.0 ml/min and 2.4 ml/min, respectively, *p* < 0.05, Table 2). Four patients in the OLP group (0.52 ± 0.045 ml/min), and one participant (0.5 ml/min) in the age- and gender-matched control group had chew-stimulated salivary flow rate of ≤ 0.7 ml/min (Table 2).

**Table 2:** Salivary flow rate (ml/min), prevalence of hyposalivation (≤ 0.7 ml/min), concentration (µg/ml) and output (µg/min) of acidic glycoproteins, total protein and calcium presented as mean ± SEM values

	OLP group	Age- and gender-matched control group	Younger control group
Chew-stimulated flow rate (ml/min)	1.5 ± 0.17*	2.0 ± 0.2	2.4 ± 0.1
Number of participants with stimulated flow rate ≤ 0.7 min	4 (0.52 ± 0.045 ml/min)	1 (0.5 ml/min)	None
Concentration of acidic glycoproteins (µg/ml)	15.4 ± 1.5**	23.1 ± 1.9	19.1 ± 1.4
Output of acidic glycoproteins (µg/min)	21.8 ± 2.4***	43.0 ± 2.9	45.6 ± 3.7
Concentration of total protein (mg/ml)	3.79 ± 0.5*	2.68 ± 0.26	2.5 ± 0.18
Output of total protein (mg/min)	5.17 ± 0.7	5.33 ± 0.68	5.48 ± 0.79
Concentration of Calcium (mM)	0.30 ± 0.03	0.33 ± 0.05	0.31 ± 0.02
Output of Calcium (mM/min)	1.21 ± 0.16	1.27 ± 0.10	1.33 ± 0.16

Significant differences between the OLP group and the age- and gender-matched control group * = *p* < 0.05, * = *p* < 0.01, *** = *p* < 0.001. No statistically significant differences were detected between the age- and gender-matched and the younger control groups

### Total protein concentration and output

OLP group showed significantly higher protein concentration (3.79 ± 0.50 mg/ml) compared to the age- and gender-matched controls (2.68 ± 0.26 mg/ml, *p* = 0.02, Table 2). However, the total protein output was slightly lower in the OLP group (5.17 ± 0.70 mg/min) compared to the age- and gender-matched controls (5.33 ± 0.68 mg/min), but this difference was not statistically significant (*p* = 0.4).

### Calcium concentration in stimulated saliva

OLP group showed a slightly lower calcium concentration (0.30 ± 0.03 mM) compared to the age- and gender-matched controls (0.33 ± 0.05 mM), but this difference was not statistically significant (*p* = 0.46, Table 2).

The total calcium output was slightly lower in the OLP group (1.21 ± 0.16 mM/min) compared to the age- and gender-matched controls (1.27 ± 0.10 mM/min), and the younger control group (1.33 ± 0.16 mM/min), but this difference was not statistically significant (*p* = 0.38, *p* = 0.37 respectively).

### Sulfated and sialylated (acidic) glycoproteins

The intra-assay coefficient of variation between glycoprotein analyses for three replicates of twelve saliva samples was 10.3%. This value falls within the range considered indicative of a reliable method [35].

The concentration of sulfated and sialylated glycoproteins in the saliva of OLP patients (15.4 ± 1.5 µg/ml) was significantly lower than that in age- and gender-matched controls (23.1 ± 1.9 µg/ml, *p* = 0.005, Table 2).

To account for the variation in flow rates, the total secretory output (µg/min) of acidic glycoproteins was calculated by multiplying the concentration of acidic glycoproteins by the respective individual's flow rate [34]. The secretory output for the OLP group (21.8 ± 2.4 µg/min) also showed a significant decrease in sulfated and sialylated glycoproteins compared to the age- and gender-matched controls (43.0 ± 2.9 µg/min, *p* = 0.0002, Table 2).

### Effect of age on the level of total protein, calcium, and sulfated and sialylated glycoproteins

In the younger control group, the protein concentration (2.50 ± 0.18 mg/ml) and total protein output (5.48 ± 0.79 mg/min) did not show any significant differences compared to the age- and gender-matched controls (2.68 ± 0.26 mg/ml and 5.33 ± 0.68 mg/min, respectively, p > 0.05, Table 2).

The calcium concentration was similar in both the younger control group (0.31 ± 0.02 mM) and the age- and gender-matched controls (0.33 ± 0.05 mM), with no significant difference observed between the two groups (Table 2).

The concentration of sulfated and sialylated glycoproteins in the younger control group (19.1 ± 1.4 µg/ml) was not significantly different from that in the age- and gender-matched controls (23.1 ± 1.9 µg/ml, p > 0.05). Similarly, the secretory output of sulfated and sialylated glycoproteins in the younger control group (45.6 ± 3.7 µg/min) was slightly higher than that of the age- and gender-matched controls (43.0 ± 2.9 µg/min, p > 0.05, Table 2); however, it did not reach a significant difference.

## Discussion

In the present study, patients with OLP showed lower amounts of sulfated and sialylated glycoproteins in the chew-stimulated saliva compared with the controls. The reason why these glycoproteins are low in the OLP group is currently unknown [36]. However, patients with OLP often complain of oral dryness [37]. In line with previous studies, the present study revealed a higher prevalence of subjective dry mouth (80%) in patients with OLP compared to age- and gender-matched controls (48%) [23, 37–39]. The low salivary flow rate among OLP patients has previously been linked to a decrease in the expression of muscarinic receptors in the salivary glands [19], or the side effects of medications [23]. Agha-Hosseini et al., [19] has shown that both unstimulated and stimulated salivary flow rates were lower in patients with OLP compared to healthy controls, which correlates with the results of the present study. However, in other studies only unstimulated but not stimulated salivary flow rates have been shown to be lower in patients with OLP [23, 24, 39]. The differences found could be due to the small number of patients included in the studies as well as the high variation of stimulated salivary flow rates between individuals. In the current study, patients included in the OLP group had a relatively high degree of oral symptoms with a high prevalence of fungal infections, which might be another explanation for the low flow rate of stimulated saliva. The secretion of water and proteins in the salivary glands is primarily controlled by the parasympathetic and sympathetic nerves of the autonomic nervous system [40]. In accordance with that, the low salivary flow rate found in patients with OLP may represent a low secretory activity of the salivary glands, which as a result may result in a low glycoprotein secretion into saliva. There was also a slight decrease in calcium concentration and total protein output, but this decrease was not statistically significant. However, further research is needed to identify the factors associated with the low glycoproteins found in patients with OLP.

The main mucin types found in human saliva are MUC5B and MUC7. Several investigations have demonstrated that glycoproteins by binding sites interact with oral microorganisms and prevent pathogen adhesion to the underlying oral epithelial cells [8]. Sulfate and sialic acid residues in the glycoprotein structure play a vital role in mucins by protecting the oral cavity from foreign microorganisms and contributing to an oral microbiome associated with oral health and disease prevention [8]. There is evidence indicating that the inflammatory process in OLP alters the glycosylation of glycoproteins, which may play a role in its pathogenesis [36]. The presence of sulfate and sialic acid residues in mucins provides protection against enzymatic degradation by bacteria or host proteases [6, 41]. In the current study, patients with OLP had low levels of sulfated and sialylated glycoproteins in chew-stimulated saliva, which may potentially lead to impaired protection against the hostile environment of the oral cavity. However, the role of the salivary sulfated and sialylated glycoproteins in the oral mucosa and their association with OLP requires further research.

OLP is a chronic inflammatory oral mucosal disease that primarily affects the buccal mucosa, tongue, and gingiva. While the exact cause is unknown, both antigen-specific and non-specific mechanisms are suggested in its development [18]. Previous studies have found a decrease in the level of salivary MUC5B, a mucin abundant in sulfated and sialylated glycoproteins [20, 42]. Moreover, the low levels of MUC5B observed in unstimulated saliva have been associated with the high levels of pro-inflammatory cytokines found in the serum of patients with OLP [20]. We did not correlate levels of salivary mucins (MUC5B or MUC7) with the levels of pro-inflammatory cytokines in serum of patients with OLP. However, our findings might support these results since MUC5B and MUC7 are rich in sulfated and sialylated glycoproteins.

Salivary mucins are heavily glycosylated glycoproteins that form a protective layer on the surfaces of oral mucosa [8]. The strong anionic character of mucins strengthens their interaction with hard and soft tissues [3]. The presence of sulfate and sialic acid groups provides protective properties to these glycoproteins [4, 6]. In addition, mucins play important roles in signalling to the oral epithelium [3, 6]. All these characteristics of mucins have an impact on the physicochemical and functional properties of saliva. The current study, along with previous studies, indicates that the presence of sulfated and sialylated glycoproteins in the oral cavity is essential for preserving the integrity of the oral epithelium in the face of challenging environments [3, 4, 6].

The disturbances in calcium responses have been associated with several autoimmune and inflammatory diseases [43]. The rise in intracellular calcium can trigger numerous cellular responses, by binding to numerous calcium-sensitive effector proteins [10]. In oral epithelium, connexin 43, the main protein associated with cellular gap junctions is related to the activation of calcium channels [44]. Cells can access two sources of calcium to generate signals: Calcium release from intracellular stores and calcium influx from the extracellular space [10, 14]. The elevation in calcium levels from either intracellular and/or extracellular stores seems to take part in the regulation of human beta-defensin 2 expressions in gingival epithelial cells [11]. A previous study found that patients with OLP had lower intracellular calcium levels in peripheral lymphocytes [25]. However, the free calcium levels in chew-stimulated saliva measured in the present study were similar in both the OLP and control groups. Our findings are consistent with another study, which also reported no significant differences in calcium levels in unstimulated saliva among OLP patients [26]. It is important to note that the calcium concentration measured in saliva may not accurately reflect the levels of calcium within the oral mucosa. This is because the method used in our study measures the levels of free calcium (dissolved calcium) in saliva, not intracellular calcium. To assess any disruptions in calcium activity in patients with OLP, it may be more appropriate to evaluate the total calcium levels in saliva, which include both free and bound calcium that interacts with salivary proteins. Further research is needed to identify the role of acidic glycoproteins in relation to calcium levels (intra- or extra-cellular source) in OLP.

Quantitative measurements of salivary mucins can be challenging due to their high molecular mass and complex structure, which form complexes with other salivary proteins [34, 45]. Therefore, different separation methods, such as electrophoresis and mass spectrometry, are required prior to quantifying glycoproteins [28]. Acidic glycoproteins are polyanionic and contain both sialic acid and sulfate residues attached to the oligosaccharide structures [6]. At acidic pH levels, these structures interact with Alcian Blue and form blue-stained precipitated complexes with both sulfated and sialic acid containing glycoproteins [46]. Alcian Blue is a sensitive technique, previously used for quantitative measurement of acidic glycoproteins in the intestines [30]. To the best of our knowledge, this is the first study using the Alcian Blue method for quantitative measurements of sulfated and sialylated glycoproteins in saliva. Based on our findings, we propose the Alcian Blue method as a reliable and simple method, for measuring sulfated and sialylated glycoproteins in saliva. Our results indicate a low intra-assay variation of 10.3%, which falls within the optimal range of values (< 20%) [35].

The participants included in the OLP group and the age and gender-matched control group were over 60 years old and showed a high prevalence in the use of medications. There has yet to be shown if there are any age-related or medication-induced differences in the levels of acidic glycoproteins, total proteins, and calcium in chew-stimulated saliva. Therefore, a healthy younger control group was included. However, there were no differences observed in the levels of sialylated and sulfated glycoproteins, total protein, and calcium between the age- and gender-matched control group and the younger control group. It is worth noting that medication intake has a strong association with both objective and subjective dry mouth [31, 47]. Based on the findings of this study, the low salivary flow rate is likely due to the use of medications, which may have contributed to the low levels of salivary acidic glycoproteins in patients with OLP. However, to confirm these findings, a larger study population would be necessary.

Limitations of the study are that only stimulated saliva samples were studied, and xerostomia was assessed by asking a single-item question. Unstimulated saliva samples with a validated questionnaire such as Xerostomia Inventory XI could be used to give a better understanding of the objective (hyposalivation) and subjective (xerostomia) dry mouth in patients with OLP. Moreover, Mucins, MUC5B, and MUC7 are rich in unstimulated saliva samples. In future studies, it would be interesting to compare the level of acidic glycoproteins between stimulated and unstimulated saliva samples in patients with OLP.

Previous studies have shown altered regulation of mucins in diseases of the gastrointestinal system, such as Crohn’s disease and ulcerative colitis. Reduced or lost sulfation of mucins has been reported in colorectal cancer [48]. Studies of patients suffering from cystic fibrosis, bronchitis and otitis media showed increased acidic-type mucins, which may be due to mucosal inflammation [49–51]. As a result, some diseases may cause aberrant mucin gene expression causing alteration in the amounts and structure of the glycoproteins. It is still unknown if reduced acidic glycoproteins initiate the lesions in OLP. When the mucus barrier is reduced or altered it may be a way for microorganisms to penetrate the underlying epithelial cells causing inflammatory responses [52], which is suggested to be the case for the OLP group in the present study.

## Conclusions

OLP patients have a high prevalence of dry mouth with low levels of acidic glycoproteins in chew-stimulated saliva. Further research is required to identify the role of acidic proteins and their association with OLP. Saliva has significant potential as a valuable research material for investigating the pathogenesis of oral diseases. The current study suggests a standardised method using Alcian blue to quantitatively measure acidic glycoproteins in saliva. In the future, studying the role of acidic glycoproteins in premalignant and malignant oral diseases may contribute to the development of novel diagnostic tools and treatment approaches.

## Data Availability

The data that support the findings of this study are available on request from the corresponding author. The data are not publicly available due to ethical restrictions.
